# Online and Face-to-Face Performance on Two Cognitive Tasks in Children With Williams Syndrome

**DOI:** 10.3389/fpsyg.2020.594465

**Published:** 2021-02-04

**Authors:** Maria Ashworth, Olympia Palikara, Elizabeth Burchell, Harry Purser, Dritan Nikolla, Jo Van Herwegen

**Affiliations:** ^1^Department of Psychology and Human Development, UCL Institute of Education, University College London, London, United Kingdom; ^2^Department of Education Studies, University of Warwick, Coventry, United Kingdom; ^3^Department of Psychological Sciences, Birkbeck, University of London, London, United Kingdom; ^4^Department of Psychology, Nottingham Trent University, Nottingham, United Kingdom; ^5^Department of Psychology, Kingston University, Kingston upon Thames, United Kingdom

**Keywords:** cognitive assessment, online assessment, face-to-face assessment, Williams syndrome, Raven’s Colored Progressive Matrices, British Picture Vocabulary Scale 3 (BPVS3)

## Abstract

There has been an increase in cognitive assessment via the Internet, especially since the coronavirus disease 2019 surged the need for remote psychological assessment. This is the first study to investigate the appropriability of conducting cognitive assessments online with children with a neurodevelopmental condition and intellectual disability, namely, Williams syndrome. This study compared Raven’s Colored Progressive Matrices (RCPM) and British Picture Vocabulary Scale (BPVS) scores from two different groups of children with WS age 10–11 years who were assessed online (*n* = 14) or face-to-face (RCPM *n* = 12; BPVS *n* = 24). Bayesian *t*-tests showed that children’s RCPM scores were similar across testing conditions, but suggested BPVS scores were higher for participants assessed online. The differences between task protocols are discussed in line with these findings, as well as the implications for neurodevelopmental research.

## Introduction

There has been an increase in conducting psychological studies and assessments online as the Internet presents a number of opportunities and advantages for academic research (Lefever et al., 2007; Lochner, 2016). The pandemic related to coronavirus disease 2019 (COVID-19) has also affected the need for online data collection as social distancing measures prevent the majority of face-to-face research to take place with little indication about when it may resume. The impact of COVID-19 and social distancing measures may be particularly relevant and long-lasting for neurodevelopmental research because the high rates of co-occurring, complex medical needs for people with neurodevelopmental conditions (Thapar et al., 2017) means face-to-face research could be especially high risk for such groups, so may not resume for some time. In addition, more psychological assessments have been undertaken via online methods in response to the COVID-19 pandemic (British Psychological Society (BPS), 2020). As such, information and evidence about the appropriability of online research for people with neurodevelopmental conditions is particularly pertinent. Online data collection is a cost-effective way of gathering data, requiring less time, and economic investment for both the participant and the researcher (Tuten et al., 2002). This can also increase participants’ response speed for taking part (Deutskens et al., 2004), making for more efficient research. Relatedly, online research can recruit larger sample sizes compared to traditional laboratory or survey methods (Wilhelm and McKnight, 2000), which in turn have higher statistical power for more reliable, generalizable interpretations (Reips, 2000; Birnbaum, 2004). Finally, the Internet allows researchers to access participants across a wider geographical area (Tuten et al., 2002) which can be particularly valuable when recruiting a specialized sample (Kraut et al., 2004), for example people with rare neurodevelopmental conditions such as Williams syndrome (WS). However, seeing the uneven cognitive and behavioral profile, intellectual impairments and limited use of technology in WS, it is unclear what the benefits and difficulties might be when assessing children with WS online. The current study is the first to compare the scores of children with WS who completed cognitive assessments online with children assessed face-to-face.

Cognitive research has become increasingly Internet based. Researchers have set up websites that host various online cognitive assessments for face and emotion recognition, implicit attitudes, and personality traits (Germine L. et al., 2011; Germine L.T. et al., 2011; Soto et al., 2011). Qualitative evidence has shown that web-based and laboratory based cognitive tests measure the same psychological constructs (Krantz et al., 1997; Birnbaum, 2000; Krantz and Dalal, 2000) and the data from the aforementioned online studies were reliable, replicable, and theoretically consistent (Germine et al., 2012). Moreover, Germine et al. (2012) established that face recognition, emotion processing, and visual memory tasks completed online had comparable mean, variance, and internal reliability performance scores to traditional, laboratory-based data. This evidence refutes the idea that online assessments produce noisier data (Kraut et al., 2004), and suggests web-based cognitive assessments do not reduce data quality. This is encouraging evidence to support the use of online testing for psychological research. However, these data were drawn from neurotypical participants without intellectual disability (ID) who had a sufficient skillset to access and use a computer and the Internet independently. Difficulties in areas such as reading and understanding, following instruction, memory, and attention can mean many people with IDs cannot access or use a computer and the Internet in the same way a neurotypical person can (Dobransky and Hargittai, 2006). As such, Germine et al.’s (2012) conclusions about convergent validity and data quality may not be generalizable to neurodivergent populations.

There is limited literature about conducting online research with people with neurodevelopmental conditions, and to our knowledge, the research that has been conducted has been restricted to autistic participants without ID. For example, Sucksmith et al. (2013) asked adults without an ID to complete an emotional faces task online, and argued that their results may be more valid than those obtained by face-to-face participation, because participants were less stressed as they could complete the task at home. Griffiths et al. (2019) were the first to examine face and emotion recognition in autistic young people (*M*_*age*_ = 11.24 years, SD_*age*_ = 2.91) by hosting cognitive tasks on a website for participants to complete with the assistance of an adult. Moreover, this study produced data that were in line with results from a meta-analysis of similar laboratory-based studies (Uljarevic and Hamilton, 2013) and added to the literature about emotion recognition in autism. Drawing from this, online testing may be appropriate for autistic young people without an ID. However, this limited research is not generalizable to provide good evidence about how appropriate online testing is for neurodivergent people with an ID, such as people with WS.

Williams syndrome is a rare congenital condition caused by the microdeletion of 26 genes on the long arm of chromosome 7 (7q11.23), and occurs in approximately 1 in 20,000 live-births (Martens et al., 2008). People with WS have mild to moderate IDs with IQs ranging from 42 to 68 (Martens et al., 2008). While there is a similar variability to a neurotypical population (Van Herwegen et al., 2011), individuals with WS have an uneven cognitive profile with areas of relative strength for auditory memory and language, but relatively weak executive functioning for attention, planning, and visuospatial skills (Bellugi et al., 2000; Mervis and Klein−Tasman, 2000; Mervis et al., 2003; Thomas et al., 2011). Again, while there are individual differences to account for, people with WS are often friendly, sociable, and even hyper-sociable (Jones et al., 2000). People with WS can have behavioral difficulties with interpersonal relationships, as well as experiencing significant issues with mental health, anxiety, and hyperactivity (Riby et al., 2014). The uneven cognitive and behavioral phenotype of WS has been of interest to researchers in neuroscience and psychology, examining biological, cognitive, and social behavior aspects of development for the past 40 years (Howlin et al., 1998, 2009).

Standardized cognitive assessments are regularly included within neurodevelopmental research for a reference of exclusion criteria, matching groups, and using as a covariate in statistical analysis (Purser and Van Herwegen, 2016). Due to uneven cognitive profiles, it is important to consider various areas of cognitive ability for people with different neurodevelopmental conditions. For example, children with WS have diverging verbal and non-verbal abilities, developing better verbal abilities more rapidly compared to non-verbal abilities (Jarrold et al., 2001). Raven’s Colored Progressive Matrices (RCPM) and the British Picture Vocabulary Scale (BPVS) are standardized aptitude assessments with excellent psychometric properties that measure non-verbal and verbal cognitive ability, respectively (Raven et al., 1990; Dunn et al., 2009). RCPM and BPVS are commonly used in neurodevelopmental research, including studies with children with WS (Jarrold et al., 2001; Mann and Walker, 2003; Farran et al., 2011; Purser et al., 2015). Both RCPM and BPVS have been adapted for computer and web-based use with typical populations (e.g., Morrison et al., 2015). However, to our knowledge there is no literature that has conducted RCPM or BPVS assessments online with children with neurodevelopmental conditions, nor evaluated the appropriability of doing so. Considering the frequency of use of RCPM and BPVS in neurodevelopmental research and the advantages of online testing, evidence supporting online RCPM and BPVS assessments with neurodivergent participants would be beneficial for the academic community, as well as the communities that benefit from the impact of research.

Due to the rarity of WS, sample sizes for psychological research in this condition are often small (Martens et al., 2008; Van Herwegen and Simms, 2020). Online research could offer WS researchers access to participants across a wider geographical area, in turn boosting studies’ sample sizes and statistical power. In addition, online research may be particularly appealing for people with WS, considering their cognitive and behavioral characteristics. Many children with WS have higher levels of anxiety and sensory needs, so completing cognitive assessments online in a familiar setting adjusted to the child’s sensory preferences may be less anxiety provoking compared to traveling to a University lab to be assessed with an unknown adult.

Nevertheless, there are a number of limitations and ethical considerations about testing children with WS online. First, online assessment could impede children with WS’ ability to complete cognitive assessments due to particular areas of need. For example, many children with WS experience difficulty with concentration and sustained attention. Without a researcher physically present to engage and redirect the child’s attention where necessary, online assessment could exacerbate this challenge and lead children to lose motivation and focus to complete the tasks. People with WS are also often highly sociable and seek to engage and interact with others (Jones et al., 2000; Doyle et al., 2004), and can also become fixated looking at people’s faces (Doherty-Sneddon et al., 2009; Riby et al., 2011). Considering the social component of assessment, the sociability of the WS cognitive profile could lead to distraction for some individuals whereby the child attempts to engage in conversation with the researcher, or becomes fixated on the researcher’s face instead of completing the task. Distractibility due to sociability may change depending on assessment context. For example, children with WS may be less distracted by the social element of assessment online because the researcher is less prominent to the child in an online set-up. However, this brings into question which are the “true” scores if results vary depending on the assessment context. This highlights the need for a comparison of assessment scores across different contexts to investigate whether scores do vary, and further elucidate whether or where an issue about true scores might lie. Due to the child’s lower IQ, a parent or carer would need to assist the child with the technological aspects throughout the assessment. This can result in a loss of researcher control over standardization of testing conditions, which can negatively impact the quality and reliability of the research (Kraut et al., 2004). Having said this, research investigating the appropriability of a Parent-Administered Neurodevelopmental Assessment for infants with Down syndrome found that with support, parents were able to gather high-quality, research-level data (Kelleher et al., 2020), suggesting a loss of direct researcher input replaced by parental/carer involvement is not necessarily detrimental to data quality and reliability. In addition, although online testing enables access to a wider population, samples are still not entirely representative as participants must have access to a computer and the Internet, as well as the ability to use both (Gosling and Johnson, 2010). Due to a *digital divide*, participants from lower socioeconomic backgrounds often have little or no access to a computer and the Internet (van Dijk, 2006; Zillien and Hargittai, 2009) and may not be included in online research. Although the advantages and disadvantages of online research are well documented, it is unclear how they relate to online research in WS.

The current study aimed to evaluate the appropriability and comparability of conducting online cognitive assessments, namely RCPM and BPVS, with children with WS. Raw scores of RCPM and BPVS assessments completed online were compared to an age-matched group of participants with WS that completed RCPM and/or BPVS assessments face-to-face. The face-to-face data was taken from the largest data-repository collection of RCPM and BPVS data in the United Kingdom (WiSDom) (Van Herwegen et al., 2019), similar to how Germine et al. (2012) collected their data to compare online and lab-based cognitive assessments with autistic people. This is the first study, conducted in 2018–2019, to use online assessment with children with WS to offer evidence about the comparability of data between online and laboratory testing for children with neurodevelopmental conditions and IDs. This evidence is especially valuable in light of the COVID-19 pandemic which could have long-term impact for face-to-face neurodevelopmental research. In line with literature showing data collected by online methods are comparable to data collected by traditional methods (Germine et al., 2012; Griffiths et al., 2019), we hypothesized that there would be no significant difference in RCPM or BPVS raw scores between the children tested face-to-face and those tested online.

## Methods

### Participants

Data from 40 participants was pooled from two different studies, including a total of 26 participants tested face-to-face (39% females) and 14 participants tested online (57% females). See Table 1 for a breakdown of participants’ chronological and mental age (MA) by assessment method and type (i.e., online or face-to-face and RCPM and BPVS scores). All participants had received a confirmed genetic diagnosis of WS. Participants included were from a narrow chronological age range from 10 to 11 years old. This restricted age range was deliberate in order to limit the impact of age as a variable that may contribute to variability in outcomes, as previous studies have shown that large variability in outcomes is often caused by the wide range of ages that are often necessary to include in WS research due to the syndrome’s rarity (Van Herwegen et al., 2011). In addition, as these standardized tasks should not be repeatedly used within a time frame of 6 months, two different samples of children were used.

**TABLE 1:** Number (*n*) of participants; Mean (Standard Deviation) of Chronological Age and Mental Age in Years (age range) of children assessed online and face-to-face for Ravens Colored Progressive Matrices (RCPM) and British Picture Vocabulary Scales (BPVS).

	RCPM (*n* = 26)			BPVS (*n* = 38)		
		
	*n*	*M* (SD) chronological age	*M* (SD) mental age	*n*	*M* (SD) chronological age	*M* (SD) mental age
Online	14	11.29 (0.39) [10.76–11.76]	5.88 (1.34) [3.6–8.24]	14	11.29 (0.39) [10.76–11.76]	6.36 (1.04) [4.92–8.25]
Face-to-face	12	11.35 (0.30) [11.00–11.75]	5.80 (1.61) [3.3–9.2]	24	11.28 (0.31) [10.83–11.81]	5.65 (1.57) [2.17–9.75]

Online participants were tested with RCPM and BPVS in 2018–2019 as part of an online research project that examined transition from primary to secondary school for children with WS (this study was approved by Kingston University Ethics Committee). The inclusion criteria for this research included children with WS to be in their last year of English primary school. This narrow eligibility restricted the participant age range and limited the online sample size, however this sample size is representative of typical studies published with people with WS (Van Herwegen and Simms, 2020). Participants were recruited via The Williams Syndrome Foundation (WSF), adverts on social media and word of mouth. Such recruitment methods could mean some of the online participants had a preexisting relationship with the research lab conducting the research, and/or the research staff involved in the project; however, this is common and often unavoidable in research with rare neurodevelopmental conditions such as WS. Participants received a £10 voucher for their time. Parents provided informed consent before the child completed the study tasks and children provided verbal assent.

Data from participants tested face-to-face were drawn from a data repository of a multi-lab, longitudinal project called WiSDom: Development in Williams Syndrome (see Van Herwegen et al., 2019 for more information). The WiSDom data repository included various forms of data (including RCPM and BPVS raw scores) from participants with WS who took part in various research projects over 30 years and gave their informed consent for the data to be added to a repository and used in future research. For the current study, RCPM and/or BPVS raw scores from participants within the same age range of the participants assessed online were utilized, so participants were matched on chronological age. For the face-to-face participants, 10 of the 12 participants with an RCPM score also had a BPVS score included in the data analysis, and the remaining two participants with an RCPM score did not have a BPVS score.

### Materials and Measures

For participants that completed RCPM and BPVS face-to-face, testing was administered as per the relevant Manual’s instructions in a quiet room, either at the participant’s home, at their school, or in a University room. For participants assessed online, the task was administered as closely as possible to the relevant Manual’s instructions (i.e., the same structure and given instructions), but differences in process are described in the procedure section.

#### Raven’s Colored Progressive Matrices

RCPM (Raven et al., 1990) is a standardized test that assesses fluid, non-verbal intelligence. There were three sub-tests of 12 items, totaling 36 items. For each item a participant was shown a colored pattern that has one part missing, and the participant was asked to choose which of the six options was the missing piece. Difficulty increased with each sub-test, and participants completed all 36 items without a time limit.

#### British Picture Vocabulary Scale, Third Edition

BPVS-3 (Dunn et al., 2009) is a standardized test that assesses verbal ability, including 14 sets of 12 items, totaling 168 items and difficulty increases with each set of items. However, basal and ceiling sets were established so participants only encountered items within their critical range. Participants were shown a page with four pictures and were asked to point to the picture that the researcher verbally said. The basal set included the first set where the participant made maximum one error and the ceiling set comprised the set in which the child made more than seven errors.

#### Online Assessment Hosting Platform

The RCPM and BPVS assessments were hosted via an online platform by jsPsych (de Leeuw, 2015) a JavaScript library for running behavioral experiments in a web browser. The library provided a flexible framework for building a wide range of laboratory-like experiments that can be run online. The assessments used in this study were developed for online use based partly on jsPsych and partly on custom programming integrating a variety of free source programming languages as follows: javascript, html, css, ajax, php, and mysqli.

### Procedure

For participants assessed face-to-face, the standard procedures were followed as described above. As such, the current section will only describe the procedure for those participants that were assessed online.

Parents were sent instructions by email and offered to discuss any aspect of the procedure with the researcher by email or phone prior to the assessment. Parents were told they needed a computer with sound and access to Internet, and they should set up in a quiet room in their home. The instructions also asked that no siblings will be present, and stated that while a parent should always be present to assist with the technology aspects, they could not help their child with answers to any of the questions. The parent should always be in-frame of the camera so that the researcher will be able to monitor the parents’ influence on their child’s answers.

For the current study, a video software, Zoom©, 2020 https://zoom.us, was used to present and guide the online assessment procedure. This platform was chosen as it was a free and accessible software that required no sign-up or account on the participant’s behalf, and had a screen share facility.

The procedure and materials were piloted prior to any true assessments, and relevant or necessary modifications were made to the assessment procedures. During the piloting it became clear that although the researcher was vigilant about parental influence, there was a minimal amount of control that the researcher could exert over how the parent was selecting answers. As such, the protocol was adapted whereby the researcher controlled the testing interface, and only shared the screen of the task with the participants. This also had the added benefit that children could not accidently or randomly pressed keys on the device. The assessments were conducted by a trained developmental psychology researcher with good experience of assessing children with WS.

To begin, the researcher logged into the RCPM and BPVS hosting website to open the tasks. Prior to the assessment the researcher emailed the parent a link which the parent could follow to enter the video call. Once a Zoom© connection had been established, the researcher talked to the participant through the tasks, read the instructions or test items, and controlled the interface and selected the answer on behalf of the child. However, as the researcher was not able to see which item the child had pointed to, the parent was asked to tell the researcher which number item the child had pointed to (each picture had either “1,” “2,” “3,” “4,” “5,” or “6” below it for RCPM, or “1,” “2,” “3,” or “4” for BPVS) and the researcher would select that answer, or, if the child was able to, they could tell the researcher the number themselves and accuracy was confirmed with the parent. The researcher was still visible in the video feed in a smaller box in the corner of the screen as the stimuli were shared and the assessment was administered. BPVS was completed first because it assessed an area of relative strength for the participants with WS (verbal abilities), which also facilitated building rapport between the researcher and participant. For BPVS online assessment, the researcher started at set four as per the BPVS manual’s guidance of a starting set for children with a MA of 5–6 years old (which is the average MA of the current sample). The RCPM was completed after the BPVS. Time taken to complete the task was highly dependent on the child and their concentration and engagement with the task, and breaks were taken as often and frequently as required to support good focus for all the tasks (although breaks were often only needed between tasks, not within task administration).

### Statistical Analyses

In this study the null hypotheses were tested as there were no differences expected between children’s raw scores for online compared to face-to-face assessment. As such, Bayesian analyses using JASP software (JASP Team, 2019) were deployed in order to establish a better understanding of the magnitude of evidence for the null hypotheses. The cut-offs used to establish sameness for the Bayesian analysis were a Bayes Factor (BF) below 0.33 if testing H_10_ (i.e., the hypothesis that there is a difference, in this case), or a BF above 3.00 if testing H_01_ (the null hypothesis). It should be noted that *anecdotal* evidence is broadly comparable to non-significant differences in frequentist analysis. Raw scores were analyzed because the RCPM and BPVS manuals do not have standardized norms for people with developmental conditions or ID. Therefore, it would not have been relevant to conduct analyses using norms derived from raw scores because the norm may not be valid for an individual with WS and may also inflate findings.

Bayesian independent *t*-tests with participants’ chronological age as the dependent variable are used to establish any significant differences between online and face-to-face participants’ chronological age. Descriptive statistics are also used to explore the average RCPM and BPVS raw scores in online and face-to-face assessment, and violin-plots are used to examine the variability of the data.

Next, a Bayesian repeated-measures analysis of variance (ANOVA) with MA for RCPM and BPVS tasks as the repeated measures factor and assessment method (online or face-to-face) as the between-subjects factor is conducted to examine and compare the cognitive profiles of the online and face-to-face participants. For the Bayesian repeated-measures ANOVA only, MA (instead of raw score) derived from either the RCPM or BPVS raw score will be used as the repeated-measures factor. This is because it would not be meaningful to compare raw scores between RCPM and BPVS as the scores represent different concepts and the measures have different scales and ranges. Therefore, using MA allows for a standardized comparison between performance on RCPM and BPVS.

Finally, two Bayesian independent *t-*tests with participants’ RCPM or BPVS raw score as the dependent variable are conducted to investigate the current research question and determine whether RCPM raw scores and BPVS raw scores are similar in online verses face-to-face assessment.

## Results

First, Bayesian independent *t*-tests were conducted to determine whether participants in the online and face-to-face conditions were matched for chronological age when they completed RCPM and BPVS. A Bayesian independent *t*-test of participants’ age at completing RCPM online or face-to-face gave a BF_10_ of 0.386, indicating anecdotal evidence that participants were of similar age. A Bayesian independent *t*-test of participants’ age at completing BPVS online or face-to-face gave a BF_10_ of 0.325, indicating moderate evidence that participants were of similar age. As such, the evidence suggests that participants were better matched for age on the BPVS task compared to the RCPM task, but would be considered as matched in frequentist analysis.

**FIGURE 1:**
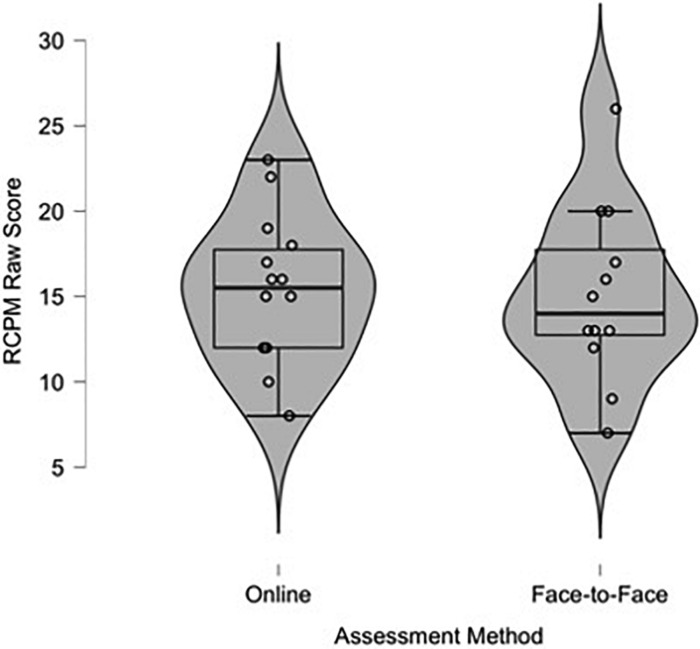
Violin-plot of children’s raw Raven’s Colored Progressive Matrices (RCPM) score assessed online and face-to-face.

**FIGURE 2:**
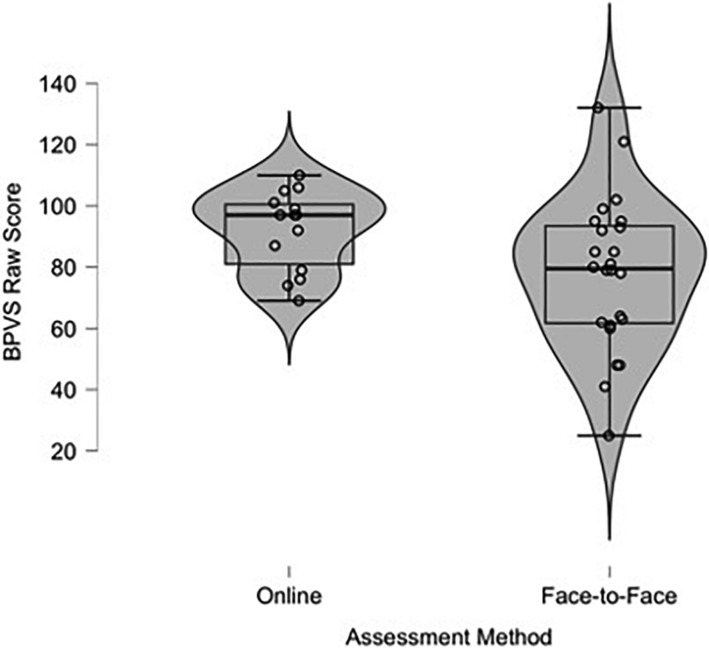
Violin-plots of children’s raw British Picture Vocabulary Scales (BPVS) score assessed online and face-to-face.

The mean RCPM raw score was 15.36 (SD = 4.33) for children assessed online, and 15.08 (SD = 5.20) for children assessed face-to-face. The mean BPVS raw score was 92.07 (SD = 13.00) for children assessed online and 77.83 (SD = 24.81) for children assessed face-to-face. Descriptive violin-plots (similar to box-plots, but with the envelope width indicating frequency in different value ranges) show the distribution and variability of children’s raw RCPM (Figure 1) and BPVS (Figure 2). For the RCPM assessments, the violin-plots show similar variability of scores. However, for the BPVS assessments there was less variability of scores among the children tested online, and more children scored lower in the face-to-face testing condition.

A Bayesian repeated measures analysis of variance with MA for RCPM and BPVS tasks as the repeated measures factor and assessment method (online or face-to-face) as the between-subjects factor was conducted in order to determine whether the participants’ cognitive profiles were similar (and therefore comparable) across assessment conditions. There was a BF_*M*_ of 2.08 in favor of the main effects model relative to a model that added the interaction term, indicating anecdotal evidence that there is no interaction between assessment method and task. Although this analysis does not provide unequivocal evidence against the interaction, the weight of evidence goes against it, therefore providing no reason to reject the notion that participants in the online assessment condition and participants in the face-to-face assessment condition had similar cognitive profiles of non-verbal and verbal IQ.

Two Bayesian independent *t*-tests were conducted in order to test the null-hypotheses that participants’ mean raw performance score for RCPM and BPVS would be similar across testing condition (i.e., whether the child completed the assessment online or face-to-face). For RCPM, analysis revealed a BF_10_ of 0.366, indicating anecdotal evidence that the scores for children with WS tested on RCPM online were similar to the scores of children that completed the task face-to-face. For BPVS, analysis revealed a BF_10_ of 1.447, indicating weak or inconclusive evidence that participants had a similar score when completing the BPVS online versus those that completed the task face-to-face. These findings do not indicate strong evidence to support our hypothesis that children will perform similarly on the cognitive tasks when assessed online compared to face-to-face, especially for the BPVS task in contrast to the RCPM task^1^. We discuss the possible reasons and implications for this in the discussion below.

## Discussion

The current study compared the RCPM and BPVS scores of children with WS from a narrow age range (10–11 years). One group of children completed these assessments online, and the second group completed the assessments via traditional face-to-face methods. In order to ensure that the two groups were matched on chronological age, RCPM and BPVS scores of age-matched participants from the largest WS data repository (WiSDom; Van Herwegen et al., 2019) in the United Kingdom were utilized for the face-to-face group. Bayesian analysis showed evidence to support the null-hypothesis that the RCPM raw scores were similar between children that completed the assessment online and those that completed it face-to-face. However, for the BPVS assessment, there was weak or inconclusive evidence for the null-hypothesis, suggesting participants assessed online scored significantly higher than children assessed in face-to-face settings. In sum, children performed similarly on the RCPM task regardless of how the testing was conducted. This suggests that viewing the patterns on screen (for which screen size may have varied across participants) or paper did not influence children’s performance. Loss of researcher control and task standardization is a key concern for online assessment, and there was potential for parents to influence children’s performance in the current study. However, the analogous RCPM scores between online and face-to-face assessment conditions suggests parents remained impartial, and allowed children to make mistakes without interfering.

There are a number of possible explanations why the BPVS scores were higher in the online assessment condition compared to the face-to-face condition. First, there may have been a recruitment bias, whereby the children that took part in the online research had particularly high verbal abilities as in the study, these participants were recruited also for required engagement in conversation about school. Second, for the online BPVS assessment the basal and ceiling items were established automatically by the computer, so scoring was less subject to human error. Multiple researchers did the face-to-face assessments, so mistakes could have been made including incorrect recording and then scoring the item, leading to different or incorrect basal and ceiling sets (although it is possible that the researchers conducting the online assessment also made errors when selecting the response). Related to this point, although the BPVS manual suggests starting points for neurotypical children, there are no recommended starting points for children with ID or WS. Often in research for children with WS, approximately half the child’s chronological age is used to gauge a child’s MA. Therefore, for online testing with children age 10–11, the researcher started at set four as this corresponded to a starting set for children with a MA of 5–6 years old. Although both online and face-to-face assessments followed the standardized administration instructions, it is possible that different starting sets were used between online and face-to-face conditions (as well as within the face-to-face condition because the scores came from different research projects in the WisDom database), which may have affected task performance and scores. Finally, assessing children with WS face-to-face could make their performance more variable as people with WS often stare at the researcher’s face during in-person assessment sessions, which can be cognitively demanding and distracting, and impact task performance (Doherty-Sneddon et al., 2009; Riby et al., 2011). This factor may have influenced performance less in the online assessment, as due to screen sharing, the researcher’s face only appeared in a corner of the screen, and therefore, explain the smaller range and variability of the testing scores. Online assessment may have been more stringent, leading to less variability in scores. Indeed, a key advantage to online research is its automated nature, so data is less likely to be affected by researcher’s error (Kraut et al., 2004). Yet, future research should compare BPVS scores from online and face-to-face assessment that follow identical procedure for starting sets in the same group of children to confirm the online advantage.

This study is the first evidence about online assessment methods for children with WS and was conducted in 2018–2019, before the impact of COVID-19 and the move to online assessment. While it is important to highlight that the findings are mixed and tentative, it is encouraging that online assessment produced similar RCPM scores compared to traditional assessment methods for a number of reasons. This is valuable initial evidence for neurodevelopmental research involving people with WS, a rare neurodevelopmental condition, particularly as the COVID-19 pandemic and social distancing measures may lead to more research having to be conducted online. Due to its rarity, recruiting adequate sample sizes can be particularly difficult for research in WS and often involves (a) great expense from traveling across the country (or even to entirely different countries) to include participants, or (b) including participants from a wide age range in order to obtain adequate sample sizes. The current study offers the first evidence that research with WS can reap the benefits of online cognitive assessment reaching a wider geographical area (Tuten et al., 2002) to maximize recruitment of a targeted participant sample, and increase sample sizes and statistical power without the necessary expense. This study can also serve as an example for the development of future online assessment for children with other neurodevelopmental conditions, especially those with mild to moderate ID and uneven cognitive profiles who experience attention difficulties. Relatedly, online testing could also benefit research in other rare neurodevelopmental conditions that suffer from similar recruitment issues such as Cri du Chat syndrome, Cornelia de Lange syndrome, and Sotos syndrome that occur 1:15,000, 1:10,000, and 1:14,000, respectively (Mainardi, 2006; Kline et al., 2007; Lane et al., 2019). The current study also provides evidence that despite their ID, children with WS were able to engage with RCPM and BPVS tasks online. This is useful evidence to suggest that some online assessment may be appropriate for children with other neurodevelopmental conditions with mild to moderate ID. Indeed, the online study also collected RCPM and BPVS data from children with Down syndrome and autistic children with and without IDs, who also engaged in the online assessment. However, without the data from a matched group of participants assessed face-to-face, it is not possible to reliably determine whether these participants performed differently having been assessed online.

Online data collection also facilitates the building of larger data repositories and the collection of longitudinal data. The current study already used the largest multi-lab data repository of WS data in the United Kingdom, WiSDom (Van Herwegen et al., 2019), collected over 30 years from various research projects. This permitted quick access to RCPM and BPVS scores of children within the same age range of those assessed online, in order to make reliable comparisons between online and face-to-face assessment. Recently, data sharing and the use of secondary data repositories have become increasingly popular in psychological research (Martone et al., 2018). Literature has cited numerous benefits, including incorporating multi-disciplinary perspectives, large cross-sectional and longitudinal data, better insight for complex questions, and improved efficacy of time and resources (Andersen et al., 2011; Gilmore, 2016). Another advantageous implication of online assessment is that data is automatically recorded and stored on an item-by-item basis, which (a) makes online data collection less prone to error by either incorrectly scoring the item or incorrectly setting the baseline or ceiling items, and (b) allows for highly detailed data to conduct more fine-grained analyses due to item-by-item data recording. The current study shows that the use of online assessments for children with neurodevelopmental conditions, especially those with WS, might provide opportunities to create larger, even international, as well as highly detailed and reliable longitudinal data repositories.

Even though the current study included only two tasks, these two tasks are the most commonly used in children with WS in the United Kingdom to assess their strengths (receptive vocabulary as is used in the BPVS), and areas of difficulties (non-verbal reasoning through RCPM). The fact that the current study shows the same pattern in cognitive profile relative to areas of strength and difficulty in a child with WS (i.e., better performance in the BPVS compared to the RCPM) in face-to-face assessments and online assessments is therefore encouraging. Nevertheless, it could be the case that some cognitive assessments that children find particularly challenging may always need to be done in person so a trained researcher can provide the appropriate support and draw out the true potential of the child’s abilities. In addition, many participants with WS have participated in cognitive or neuropsychological assessments before through research, standard of care or for educational purposes. Therefore, while it is likely that completing cognitive assessments online was novel for the current sample, these participants are often familiar with cognitive assessment procedures, which could facilitate an easier transition to online assessment compared to a child with no familiarity with cognitive assessment formats, which may affect task performance. Future research should investigate the suitability of different types of cognitive tasks in an online format according to participants’ areas of relative strength and difficulty, which may change depending on the cognitive profiles of the individual, different developmental conditions, and the amount of prior experience with cognitive assessment.

This study is not without its limitations. A first limitation to acknowledge is the small and uneven sample size for each assessment condition in the current study, which was a result of WS rarity and the restricted age inclusion criteria. The restricted age range was partly due to the fact that the online participants were part of a larger study about United Kingdom school transition from primary to secondary school for children with WS, but also to control for the fact that variability within test scores in WS are often driven by development and differences in chronological age. As such, the age range was restricted to children aged 10–11 years old. However, according the WSF United Kingdom database the online research was able to recruit a high proportion of the target population: 14 participants accounted for approximately 30% of the entire United Kingdom WS population aged 10–11 years old at the time of recruitment. In addition, although small, the current study’s sample size was still representative of typical sample sizes of studies including people with WS (Van Herwegen and Simms, 2020). A second related limitation is that the current study’s restricted age range limits the generalizability of the current findings. For example, online assessment may not be appropriate for younger children with WS with a lower MA. Future research should conduct online assessment with a wider age range and bigger sample size to establish the appropriability and validity of online assessment for different age groups.

In conclusion, this study provides the first evidence about conducting cognitive assessments online for children with WS age 10–11 years. While online assessment produced higher BPVS scores compared to face-to-face assessment, findings provided anecdotal evidence that conducting RCPM via the Internet produces analogous scores for children with WS compared to completing the tasks traditionally in face-to-face settings. This supports previous research that online cognitive assessment does not necessarily lead to poor quality data for all assessments (Germine et al., 2012), and demonstrates a good example for development of online assessment for other neurodevelopmental conditions with uneven cognitive profiles and attention difficulties. Although the current study was conducted before COVID-19, the evidence has important implications, especially considering social distancing measures due to COVID-19 mean assessment for research and schooling increasingly move online, and children and families’ comfort with online assessment increases too. The current findings provide an opportunity for neurodevelopmental research to build upon and benefit from the numerous advantages of online assessment.

## Data Availability Statement

The datasets presented in this article are not readily available because when the online data was collected, participants did not give permission for the data to be uploaded or shared to third parties. Therefore, we would need to ask participants’ permission first before sharing any data. Requests to access the datasets should be directed to JV, j.vanherwegen@ucl.ac.uk.

## Ethics Statement

The studies involving human participants were reviewed and approved by the Kingston University Faculty Research Ethics Committee. Written informed consent to participate in this study was provided by the participants’ legal guardian/next of kin and children provided verbal assent.

## Author Contributions

All authors has made a substantial contribution to the research and drafting the manuscript. JV, OP, and HP conceived the idea for the manuscript. DN set up the online assessment’s hosting platforms with input from JV and MA. MA and EB conducted the online assessment with oversight from JV and OP throughout. MA and HP conducted the statistical analyses. MA drafted the manuscript with oversight and input throughout from JV, OP, EB, HP, and DN.

## Conflict of Interest

The authors declare that the research was conducted in the absence of any commercial or financial relationships that could be construed as a potential conflict of interest.

## Footnotes

1 Conventional independent *t*-tests did not differ significantly between assessment conditions for RCPM (*t*(24) = 0.147, *p* = 0.885, *d* = 0.058) or BPVS (*t*(36) = 1.986, *p* = 0.055, *d* = 0.668) raw scores.
